# Understanding and addressing populations whose prior experience has led to mistrust in healthcare

**DOI:** 10.1186/s13584-023-00565-w

**Published:** 2023-04-21

**Authors:** Dan Even, Shifra Shvarts

**Affiliations:** Moshe Prywes Center for Medical Education, Faculty of Health Sciences, Ben-Gurion University of the Negev, P. O. Box 653, 8410501 Beer-Sheva, Israel

**Keywords:** Public trust, Accountability, Clarity of responsibility/ liability, Health messaging, Disclosure, Radiation treatments, Reparations, Population health

## Abstract

**Background:**

Policy makers need to maintain public trust in healthcare systems in order to foster citizen engagement in recommended behaviors and treatments. The importance of such commitment has been highlighted by the recent COVID-19 pandemic. Central to public trust is the extent of the accountability of health authorities held responsible for long-term effects of past treatments. This paper addresses the topic of manifestations of trust among patients damaged by radiation treatments for ringworm.

**Methods:**

For this mixed-methods case study (quan/qual), we sampled 600 files of Israeli patients submitting claims to the National Center for Compensation of Scalp Ringworm Victims in the years 1995–2014, following damage from radiation treatments received between 1946 and 1960 in Israel and/or abroad. Qualitative data were analyzed with descriptive statistics, and correlations were analyzed with chi-square tests. Verbal data were analyzed by the use of systematic content analysis.

**Results:**

Among 527 patients whose files were included in the final analysis, 42% held authorities responsible. Assigning responsibility to authorities was more prevalent among claimants born in Israel than among those born and treated abroad (*χ*^2^ = 6.613, df = 1, *p* = 0.01), claimants reporting trauma (*χ*^2^ = 4.864, df = 1, *p* = 0.027), and claimants living in central cities compared with those in suburban areas (*χ*^2^ = 18.859, df = 6, *p* < 0.01). Men, younger claimants, patients with a psychiatric diagnosis, and patients from minority populations expressed mistrust in health regulators.

**Conclusions:**

Examining populations' perceived trust in healthcare institutions and tailoring health messages to vulnerable populations can promote public trust in healthcare systems.

## Background

Effective communication with the public is vital for all national institutions and systems fostering and maintaining public health [1]. Ideally, decisions made by health organizations should be congruent with population aims and distinct societal features and enhance public willingness to attend to health messaging, accept recommendations, and adhere to treatments [2].

In recent years, public trust in health institutions has gained recognition as a highly important aspect of health policy, becoming an indicator for healthcare systems and providing governments and institutions with information on their performance from users' perspectives [3, 4]. Although the concept of public trust is difficult to define and investigate [4], recent studies in several countries have focused on patients’ trust in healthcare organizations, examining its impacts on the general public and specific populations' acceptance of health recommendations and its influence on health-promoting opinions and behaviors with relevance to public health [5–7]. In Israel, since 2016 the topic of trust in healthcare systems has been included in formal questionnaires distributed by the Central Bureau of Statistics (CBS) to assess population health [8].

The discourse on public mistrust has become more prevalent in the course of the worldwide COVID-19 pandemic outbreak [9], and it is focused on socioeconomic gaps and concerns arising from pseudo-scientific reporting and "conspiracy theories" in society [10]. For instance, Hardy et al. examined sociocultural responses to COVID-19, including politicized narratives of fear and blame that jeopardize trust in authorities' responsibility in controlling the pandemic [11].

Institutional trust is defined as the expectation that institutions will act honestly and ethically with the public [12]*.* In this era of an increasingly saturated information landscape and the rising popularity of social media, health-promotion messaging can be refined and targeted to specific populations [13] to enhance trust and adherence [14]. Currently, such population-specific adjustment of messaging regarding health guidelines and recommendations is the main method used by health authorities to maintain public trust. As a complementary step, health organizations, including government agencies, have recently used surveillance tools to assess and maintain the trust of populations in healthcare systems [15].

One determinant of public trust is the expectation that medical authorities will be considered accountable for their decisions, messages, and treatments. Such responsibility on the part of healthcare authorities is considered essential for producing, maintaining, and preserving public trust [16].

Various health organizations and ethicists, inspired by the Hippocratic Oath, have set out to define the boundaries of clinical responsibility—boundaries that include a commitment to ensuring that patients are fully informed of any known risks of treatments [17]. However, this imperative seems to apply only when treatments are given. Are medical institutions and organizations also responsible for negative effects of well-intentioned treatments which might only be known in the future? And how can such responsibility affect the public's trust?

These questions are especially relevant when iatrogenic complications emerge long after treatments. Most known cases are medications and technologies withdrawn from the market due to links to medical complications. In such cases, issuing public warnings about risks and side-effects when discovered has been found to influence public trust [18].

Weiner's Attribution theory predicts that people who attribute health issues to environmental influences, as opposed to those who believe health to be within the control of the self, tend to assign more responsibility to other entities for health issues [19]. Sociodemographic components may also contribute to accountability patterns among populations. This was demonstrated in the Tuskegee syphilis study in the United States (U.S.), causing mistrust within the Black American population toward U.S. national health institutes [20].

Within medical ethics discourse, the advancement of scientific knowledge carries with it the responsibility to ensure that institutions and clinicians report adverse events resulting from treatments and that such information will be available to patients and the public. Recent developments in medical technologies and progress in genetic treatments underscore the need to formulate a generic model for messaging problems associated with earlier treatments [21]. The increase in lawsuits concerning information duties heightens the need for health organizations to develop health communication tools and strategies for such scenarios [22]. Moreover, the new trend in healthcare systems toward patient empowerment strengthens the need to promote liability discourses within the complex therapist-patient relationship [23]. All of these factors have substantial influence over patients' trust attitudes.

Several recent studies suggest there is an emerging widespread and increasing mistrust toward health authorities and health-promotion messages. Contemporary society is defined, in part, by a general distrust in institutions and authorities [24]. Suspicion of motivations has become a default position for many, as evidenced by the strength of the belief in conspiracy theories consistent with the “cultural logic of modernity” [25]. Cancer screening, organ donation, and vaccination research each report that public trust in clinicians has been steadily decreasing in recent years [26]. This phenomenon also extends to clinicians themselves, expressed for example in limited adoption of clinical guidelines among experts [27].

Our study examines the concept of trust in health authorities as it concerns their perceived responsibility for damages in a unique scenario of ionizing radiation used to treat ringworm of the scalp decades ago.

X-ray radiation treatment were commonly used in the first half of the twentieth century, when it was believed to be a safe and advanced treatment for various diseases and medical conditions, including ear infections [28], acne scars, adenoids, and in measuring foot size [29]. The procedure was standardized in 1907 by Robert Kienböck and again in 1910 by Horatio George Adamson and became known as the Adamson-Kienböck method [30]. Irradiation ended in 1960 when an oral antifungal medication (griseofulvin) was proven effective.

X-ray treatments for ringworm were state-of-the-art practice during the first half of the twentieth century and were documented since its first description in France in 1902 [31], and around the world including in the U.S. [32, 33], England [34] and Australia [35]. Radiation treatments for ringworm were common in pre-state Israel and expanded during the years of mass immigration after the War of Independence in 1948. Ringworm was most prevalent among children who immigrated from Jewish communities in countries in Asia and North Africa and lived in overly crowded conditions with limited facilities. It is estimated that about 27,600 children from Eastern Europe were treated by irradiation for ringworm between 1921 and 1938; 31,400 Jewish and Arab children were irradiated between 1925 and 1960 in Israel; and 22,000 Jewish children were irradiated between 1947 and 1960 in Morocco before emigrating to Israel [36].

In the 1960s, research in the U.S. and Israel indicated that this treatment for scalp ringworm increased the risk of tumors [37, 38]. In Israel, findings of the late health effects of treatments, which were focused especially on immigrant populations, garnered media attention and led to passage of unique legislation in 1994. The law provides compensation for patients (or their next of kin) who were treated with irradiation for ringworm between 1946 and 1960 and were subsequently diagnosed with a primary malignant tumor of the head or neck, brain or skull, salivary glands, or thyroid, skin tumors such as melanoma on the head and neck, benign brain tumors, leukemia, and permanent hair loss (alopecia) due to scalp scars [39]. The National Center for Compensation of Scalp Ringworm Victims was established in 1995 to implement the law, set compensation, and allocate payments.

Our study explores the attitudes of patients treated with irradiation for ringworm and the extent to which they perceived the authorities to be responsible for the negative effects of treatments emerging many years later, reflecting their trust in healthcare organizations.

## Methods

We examined attitudes expressed in medical records of patients seeking compensation for harm caused by ringworm radiation treatments. The study was designed as a mixed-methods qualitative-quantitative study of claimants approaching the Israeli National Center for Compensation of Scalp Ringworm Victims.

We used a stratified purposeful sample of 600 medical files of patients claiming compensation in the years 1946–1960, who received radiation treatments in Israel and/or abroad. The sample was formed using the criteria sex and date of case submission: 300 male and 300 female plaintiffs, submitted in a period of 20 years from the opening of the compensation center in January 1995 through December 2014, stratified each year across two seasons, with a specific order: 15 first files from January each year, from 8 men and 7 women, and 15 first files from July each year, from 7 men and 8 women. The sample was drawn from a total of 45,249 patients who submitted a request for compensation under the Scalp Ringworm Compensation Law before 2015. Retrieval of data from medical files was carried out in accordance with ethical standards for such work as set forth in Article 20 (A) (7) of Israel’s 1996 Patient’s Rights Law [40]. This permits the patient or medical institution to provide medical information that is “designed for publication in scientific journals, for research purposes or teaching, in accordance to directives set forth by the Minister of Health, provided identifying details of the patient will not be revealed”.

Data in files were anonymized by the National Center staff to exclude all personal identification before analysis. The research protocol including the anonymization process was approved by the Helsinki committees of Sheba Medical Center (#2448-15-SMC) and Ben-Gurion University of the Negev (#2015 14) in Israel.

The application form and files written by the medical committees that discussed and judged the cases at the National Center for Compensation of Scalp Ringworm Victims required claimants to provide personal information; this included demographic data on sex, age at claim submission, place of residence, ethnicity, marital status, education and employment status, army service, place of birth, location of radiation treatments, and diagnosed diseases, including cancer, chronic diseases and mental diseases. Claimants were requested to specify which authorities, if any, they held responsible for the effects of treatment. Each patient’s area of residence and socioeconomic status were estimated by using the postal code of participants at the time of their legal claim submission, according to Israeli Census data [41]. Socioeconomic cluster 1 represents the lowest socioeconomic level, and cluster 10 represents the highest one.

Quantitative data were analyzed using descriptive statistics in IBM_SPSS_25, and correlations were analyzed using the chi-square test.

Verbal data concerning attitudes of patients were extracted from the files and analyzed using a qualitative method according to the principles of grounded theory and using systematic content analysis for the purpose of defining categories and subcategories. Grounded theory aims to identify themes in verbal material through persistent analysis of data to formulate arguments through a process strengthening the reliability and validity of qualitative material [42].

## Results

The sampling strategy initially identified 568 claimant files, but due to duplicate and missing data, a final sample of 264 men and 263 women was analyzed (N = 527).

On average, claimants applied to the National Center for Compensation of Scalp Ringworm Victims at 57.1 years of age (SD = 8.29 [40–88]). The majority of patients consisted of Jewish Israelis, and 5.1% (N = 27) claimants were Arabs and were categorized as the minority group (Table 1**)**.

**Table 1:** Demographic characteristics of claimants

Variable	Value	%	N
*Sex*			527
Male	264	50.1	
Female	263	49.9	
Age at claim	57.1		499
*Ethnicity*			527
Jewish	500	94.9	
Arab	27	5.1	
*Marital status*			527
Married	382	72.5	
Divorced	59	11.2	
Separated	7	1.3	
Widowed	41	7.8	
Single	25	4.7	
Unspecified	13	2.5	
*Education*			525
Up to 9 years	202	38.3	
10 years and above	323	61.3	
*Employment*			527
Unemployed	37	7	
Employed	388	73.6	
Unspecified	102	19.4	
*Army service*			526
Yes	169	32.1	
No	96	18.3	
Unknown	261	49.6	
*Residence (district)*			454
North	62	11.8	
Haifa	55	10.4	
Center	107	20.3	
Tel Aviv	93	17.6	
Jerusalem	36	6.8	
South	98	18.6	
*Socioeconomic status*			454
Cluster 2	23	4.4	
Cluster 3	48	9.1	
Cluster 4	63	12	
Cluster 5	97	18.4	
Cluster 6	74	14	
Cluster 7	76	14.4	
Cluster 8	67	12.7	
Cluster 9	6	1.1	
*Place of birth*			526
Israel	61	11.6	
Abroad	465	88.4	
*Place of Irradiation*			505
Israel	288	54.6	
Abroad	211	40	
Both	6	1.1	
*Diseases*			527
Cancer	212	40.2	
Psychiatric conditions	336	63.8	
Trauma	67	12.7	

Data extracted from files indicated that most claimants (58%) did not hold authorities responsible for the ringworm radiation treatments.

Data from the files of 221 (42%) claimants who held authorities responsible for the effects of treatment revealed that a large percentage assigned responsibility to the Israeli Ministry of Health (MOH, 25.3%) and the State itself (23.1%), as well as to organizations that inflicted radiation treatments in other countries (e.g., Morocco) before the affected individuals emigrated to Israel, including the Jewish Agency ("Hasochnut") and the American Jewish Joint Distribution Committee (or the "Joint Committee") (28.1%). Other organizations held responsible include the Israeli National Health Maintenance Organizations (HMOs—the Israeli public health insurers or health funds) (13.1%) and hospitals (5.4%). Patterns of perceived responsibility are reported in Table 2.

**Table 2:** Authorities held responsible for radiation treatments (information applies only to participants who indicated a responsible authority)

Authority	Total	
	N	%
Ministry of Health (MOH)	56	25.3
The State of Israel	51	23.1
The Jewish Agency and the Joint committee	62	28.1
Health Maintenance Organizations	29	13.1
Hospitals	12	5.4
Other	11	5
Total	221	100

Assignment of responsibility to authorities was more prevalent among claimants born in Israel than it was among those born and treated abroad (*χ*^2^ = 6.613, df = 1, *p* = 0.01); more prevalent among claimants treated in Israel than it was among those treated abroad before emigrating to Israel (*χ*^2^ = 23.855, df = 1, *p* < 0.0001); more prevalent among claimants reporting trauma (*χ*^2^ = 4.864, df = 1, *p* = 0.027); and more prevalent among claimants living in central cities in Israel (i.e., Tel Aviv and surrounding areas) than among those in other areas with suburban characteristics (*χ*^2^ = 18.859, df = 6, *p* = 0.004). Perceived responsibility did not differ significantly by socioeconomic status (*χ*^2^ = 3.572, df = 7, *p* = 0.827). Details are reported in Table 3.

**Table 3:** Factors influencing responsibility assignment for damages from radiation treatments

Factor	Assigning responsibility to authorities			
		Yes	No	Total
*Trauma*				
Yes	N	36	30	66
	%	54.5%	45.5%	100%
No	N	185	275	460
	%	40.2%	59.8%	100.0%
*p* = 0.027				
*Place of birth*				
Israel	N	35	26	61
	%	57.4%	42.6%	100%
Abroad	N	186	278	464
	%	40.1%	59.9%	100%
*p* = 0.01				
*Place of irradiation*				
Israel	N	149	139	288
	%	48.3%	51.7%	100%
Abroad	N	63	148	211
	%	29.9%	70.1%	100%
Abroad & Israel	N	4	2	6
	%	33.3%	66.7%	100%
*p* < 0.01				
*District of residence in Israel*				
North	N	18	44	62
	%	29%	71%	100%
Haifa	N	18	37	55
	%	32.7%	67.3%	100%
Center	N	55	52	107
	%	48.6%	51.4%	100%
Tel Aviv	N	51	42	93
	%	54.8%	45.2%	100%
Jerusalem	N	13	23	36
	%	36.10%	63.90%	100%
South	N	40	58	98
	%	40.8%	59.2%	100%
West Bank	N	0	3	3
	%	0%	100%	100%
*p* = < 0.01				

In a subset analysis of claimants holding authorities responsible for the effects of treatment (N = 221), claimants treated in Israel were more likely to assign responsibility for the effects of radiation treatments to organizations who functioned inside Israel, including the MOH (33.6%), the State (26.8%), and HMOs and hospitals (19.5%), whereas those treated abroad were more likely to assign responsibility to organizations operating there, such as the Jewish Agency and Joint Committee (77.8% [*χ*^2^ = 127.247, df = 15, *p* < 0.0001]) (Table 4**)**.

**Table 4:** Authorities held responsible by patients with ringworm who were damaged by radiation treatments

Authority	Responsibility among patients irradiated in Israel		Responsibility among patients irradiated abroad		Responsibility among patients irradiated In Israel and abroad	
	N	%	N	%	N	%
Ministry of Health	50	33.6	4	6.3	1	25
The State of Israel	40	26.8	10	15.9	0	0
The Jewish Agency/ The Joint Committee	9	6	49	77.8	2	50
Health Maintenance Organizations	29	19.5	0	0	0	0
Hospitals	12	8.1	0	0	0	0
Other	9	6	0	0	1	25
Total	149	100	63	100	4	100

*p* < 0.01

Claimants with a psychiatric diagnosis tended to hold the State responsible (29.1% vs. 12.5% of the patients without a psychiatric diagnosis), whereas those without a psychiatric diagnosis were more likely to assign responsibility to the MOH (32.5% vs. 21.3% of the patients with a psychiatric diagnosis [*χ*^2^ = 14.046, df = 5, *p* = 0.015]). More Arab claimants, who belong to a minority population in Israel, held the MOH responsible for the adverse effects of treatment, in comparison with Israeli Jews [*χ*^2^ = 24.443, df = 5, *p* = 0.00017], thus demonstrating mistrust in the health regulator.

Secondary analysis comparing claimants who ascribed responsibility to Israeli health authorities (e.g. MOH, HMOs, hospitals [N = 97]) versus those ascribing responsibility to the State (N = 51) and to authorities functioning abroad (N = 62) demonstrated sex differences: while male claimants (N = 114) were more likely to ascribe responsibility for treatments to "the State" (28.9% vs. 18.8% of women), women (N = 96) were more likely to hold authorities operating abroad responsible (37.5% vs. 22.8% of men [*χ*^2^ = 6.270, df = 2, *p* = 0.043]). Claimants aged 65 or younger were more likely to hold Israeli institutions responsible (50.3% vs. 26.5% of older claimants), whereas claimants older than 65 were more likely to ascribe responsibility to international agencies (44.1% vs. 25.7% of younger claimants [*χ*^2^ = 7.141, df = 2, *p* = 0.028]).

The narratives we extracted from the claimants’ files and analyzed were organized into the following six general categories of attitudes toward the ringworm treatment and its effects: suffering from the radiation treatments; compensation demands; references to DDT spraying, which was another treatment given to immigrants and residents against malaria at the time; references to metaphors related to the Jewish Holocaust; association between the radiation treatments and difficulties in broader areas of life; and anxiety about the future. Subsequently, we defined categories regarding claimant attitudes toward the level of severity of the ringworm radiation treatments: viewing the treatments as a “mess up”, a wrongdoing, a humiliating event, a “tragic event”, a traumatic event, a rape, a negligent event, torture, abuse, hell, a crime, or a murder.

## Discussion

Public health systems are societal institutions resistant to change and scrutiny, yet current governments can no longer assume that the public will believe and trust all health information and directives, and health practitioners no longer enjoy the status of "all-knowing authorities".

Our analysis allows an examination of levels of trust, that are illuminated by clarification of the assignment of responsibility of healthcare organizations in relation to radiation treatments for ringworm of the scalp later connected to harmful consequences.

The recent COVID-19 pandemic has highlighted public mistrust in health systems [9–11]. However, although both the ringworm treatment examined in our study and the COVID-19 scenario involve preventive measures to halt an expanding disease, it should be noted that the mistrust expressed by patients in our study exists in the setting of legal claims of irradiated patients for compensation and differs from healthy citizens’ mistrust of governments in regard to preventive measures against the COVID-19 pandemic.

The ringworm treatment examined in our study presents a globally unique situation, in light of the unique law compensating patients who were harmed. The affair is characterized by the lack of an organized notification process to notify patients of risks [43], which might be a contributor to the patterns of blame and mistrust revealed in our study.

The study indicates most claimants did not hold authorities responsible for the radiation treatments and the resulting harms. However, we cannot attribute wide confidence in authorities to most patients, since claimants in the study were not forced to specify their positions regarding the possible responsibility of organizations involved with radiation treatments, i.e. answering the question was not mandatory. Despite claiming compensation due to harms, subjects may have chosen not to respond to the question of responsibility for various reasons, such as misunderstanding the form and/or concern that assigning responsibility to authorities might jeopardize their claims.

However, the tendency found in our study, demonstrated by almost half of the patients harmed from radiation treatments, to blame healthcare institutions, is similar to descriptions in the scientific literature of public reactions to the public health crises. According to a cultural theory of Douglas, disasters in public health tend to develop into a political discourse in which an unpopular faction is usually blamed [44]. Eichelberger describes the politics of blaming within the SARS pandemic in the U.S., when the population of Chinese immigrants in New York was blamed for causing the disease to spread [45]. The COVID-19 pandemic was also characterized by blaming discussions, with stigma and xenophobia developing against the Chinese population for spreading the virus in countries where the Chinese are considered minorities [46].

The ringworm claimants’ mistrust in Israeli healthcare organizations might, in addition to the lack of a clear notification process, also be partly explained by the emotional influences of such treatments [47], legal claims against the Israeli government based on the duty to inform patients at risk [43], and a reluctance of the Israeli Authorities to publish a formal apology for implementing the treatment regime [48].

In comparison with immigrants, the higher tendency of patients born in Israel to hold organizations responsible for the treatments might arise from their better familiarity with the authorities, and/or it might reflect a habit among Israelis to assign responsibility in the public domain, as demonstrated by the cultural differences in responsibility perceptions toward public establishments revealed in other studies [49].

The shift toward clarity of responsibility reflecting mistrust among patients who suffered from trauma is supported by studies involving patients with post-traumatic stress disorder (PTSD) [50], who present a lack of trust in individuals and systems, including physicians and the medical system [51]. A stronger orientation toward clarity of responsibility among people residing in central compared with peripheral areas might reflect a tendency observed in studies among citizens of peripheral areas to express mistrust and assign responsibility to local entities rather than to governmental institutions [52]. The greater likelihood of younger adults (age 65 and under) to hold Israeli health authorities responsible for the treatments may reflect an increased tendency among the younger generations to criticize authorities and identify them as responsible for failures. Such a coeval tendency correlates with the findings of Yu et al. that identify accountability patterns in China toward government policies on road accidents, especially among youth [53].

Our study reveals a trend among patients from a minority group (Israeli Arab irradiated patients) to express mistrust toward official health regulators (the MOH). A similar tendency characterizes minority groups in the U.S., for example among Blacks and Mexicans expressing medical mistrust and unique responsibility attribution patterns [54, 55]. These attitudes might reflect ethnic and cultural differences and public tension between subpopulations. Several studies have examined aspects of trust in healthcare systems among minorities concerning vaccinations. In line with the results from our study, Mahmud et al. found that, compared with older U.S. white Medicare recipients, Hispanic, Black, and Asian beneficiaries were less likely to receive the recommended yearly vaccine against influenza [56]. This tendency toward mistrust among minorities might also reflect broader discrepancies between minorities and the majority population as an expression of aggravated healthcare inequality [57].

The categories of the narratives examined in claimant files correlate with other references to the ringworm case in the Israeli media and scientific literature. For example, the descriptions of patients finding similarities between the radiation treatments for ringworm and the spraying of DDT against malaria were previously described in an article claiming "some of the irradiated patients refer to the treatments (of irradiation and DDT spraying) as a deliberate humiliation" [58]. Such correlation is also mentioned in Israeli popular media publications dating back to the early 1990s, before the legal and official institutional recognition of the ringworm case [59, 60]. Israeli media publications also link radiation treatments to the Jews' history of the Holocaust; for example, in an article by Larom describing the ringworm case as the "Holocaust of North African Jews" [61]. Metaphorical correlations with the Holocaust are common in Israeli culture as part of a “Holocaust Awareness” developed throughout the years after the country's establishment [62].

### Limitations

Our study has certain limitations. First, some files in the sample lack relevant data. Moreover, data were documented in handwriting, which was sometimes illegible. Second, applicants for compensation pass before several expert committees before their requests are granted or rejected, and some files lacked uniformity regarding professionals' opinions about damages. Third, as mentioned, most claimants in the sample did not hold authorities responsible in the files, so the main analysis is based on the files of the 42% of claimants who held authorities responsible for the effects of treatment. Fourth, compensation for scalp ringworm is a sensitive topic and some applicants might have exaggerated their distress, as noted in typical discourses of public members addressing committees for injured workers [63]. Moreover, some applicants might have been directed by lawyers and other consultants, including non-profit organizations with agendas, who were managing the claims for compensation. Such interventions impact claimants' statements—for example, through overstatement of harms, due to financial or political motives [53]. The existence of a law for compensation of scalp ringworm victims puts a certain amount of responsibility—even if not legally acknowledged—on healthcare authorities, and thus may affect participants' attitudes. Thus, implications regarding scenarios of medical interventions and policies that lack legal recognition should be drawn with caution.

### Public health implications

Revealing patterns of perceived responsibility is a substantial factor in understanding public trust in health authorities and institutions, an area of growing importance in healthcare policy discourse.

Our findings emphasize the need for healthcare organizations and institutions to examine patterns of trust among specific populations in health messaging missions related to public health matters, including late hazards related to previously acceptable medical treatments, such as irradiation for ringworm and other events that require patients to be informed of discovered hazards.

This study stresses the need to adjust messages about public health events to specific populations and audiences, such as patients suffering from trauma, those living in central cities, and minorities, who are prone to certain responsibility attribution patterns, in order to enhance public trust. Recent studies emphasize the relevance and benefits of audience segmentation and tailoring messages to specified populations [64]. For instance, tailored internet-based messages were found to be beneficial for intervening with patients with mental health issues such as trauma, because they offer anonymity, reduce fear of stigma, and increase self-disclosure [65]. Rayens et al. have indicated the need to tailor smoke-free media campaigns in the U.S. to the rural populations that are more exposed to tobacco, focusing more on the benefits of smoke-free environments in gain-framed ads, as opposed to the different approaches recommended for use with urban residents [66]. Several studies offer strategies to customize health messages directed toward minorities. For example, in the U.S., Sylvester offers strategies to direct health messages and craft health campaigns for Black Americans by involving local opinion leaders and black healthcare professionals and referencing unique cultural attitudes. These include addressing fatalism and special consideration of the barriers to healthcare experienced by Black Americans, among others [67]. Kalagy et al. have demonstrated the Israeli practice of adjusting messages about the COVID-19 pandemic to religious minorities, fitting messages to the collectivist nature of the community and choosing traditional media platforms to distribute messages, in aspiring to enhance trust [68]. Our study highlights the need to further explore the types of messages most likely to work for specific subpopulations.

Developing methods to increase trust among civilians in general and patients in particular might help healthcare institutions and organizations behave more ethically and thus improve healthcare systems' functioning and reliability.

## Conclusions

Our study provides evidence of responsibility and blame discourses and patterns of mistrust among patients of specific populations toward healthcare organizations and authorities in the ringworm case, as an example of a public health case. This is a notable tendency relevant to the COVID-19 pandemic era and to challenges healthcare systems will face in the future.

The tendencies of certain populations to distrust healthcare organizations, as demonstrated in our paper, might limit these populations’ access to suitable medical care and aggravate inequalities in health systems.

Policy-makers facing such mistrust among specific populations may develop coping mechanisms based on the historic experience, which is described in our study. Our findings and their suggested implications might help national health systems and policy-makers to improve health messaging and direct it effectively toward specific populations. It is to be hoped that this will improve their credibility as well as increase satisfaction and trust among patients.

## Data Availability

The dataset used and/or analyzed in the current study is available from the corresponding author on reasonable request.

## Abbreviations

HMOs: Health maintenance organizations
MOH: Ministry of health
PTSD: Post-traumatic stress disorder
U.S.: United States
